# N-P Co-Limitation of Primary Production and Response of Arthropods to N and P in Early Primary Succession on Mount St. Helens Volcano

**DOI:** 10.1371/journal.pone.0013598

**Published:** 2010-10-26

**Authors:** John G. Bishop, Niamh B. O'Hara, Jonathan H. Titus, Jennifer L. Apple, Richard A. Gill, Louise Wynn

**Affiliations:** 1 School of Biological Sciences, Washington State University, Vancouver, Washington, United States of America; 2 Department of Biology, SUNY Fredonia, Fredonia, New York, United States of America; 3 Brigham Young University, Provo, Utah, United States of America; University of Pretoria, South Africa

## Abstract

**Background:**

The effect of low nutrient availability on plant-consumer interactions during early succession is poorly understood. The low productivity and complexity of primary successional communities are expected to limit diversity and abundance of arthropods, but few studies have examined arthropod responses to enhanced nutrient supply in this context. We investigated the effects of nitrogen (N) and phosphorus (P) addition on plant productivity and arthropod abundance on 24-yr-old soils at Mount St. Helens volcano.

**Methodology/Principal Findings:**

We measured the relative abundance of eight arthropod orders and five families in plots that received N, P, or no nutrients for 3–5 years. We also measured plant % cover, leaf %N, and plant diversity. Vegetation responded rapidly to N addition but showed a lagged response to P that, combined with evidence of increased N fixation, suggested P-limitation to N availability. After 3 yrs of fertilization, orthopterans (primarily *Anabrus simplex* (Tettigoniidae) and *Melanoplus spp* (Acrididae)) showed a striking attraction to P addition plots, while no other taxa responded to fertilization. After 5 yrs of fertilization, orthopteran density in the same plots increased 80%–130% with P addition and 40% with N. Using structural equation modeling, we show that in year 3 orthopteran abundance was associated with a P-mediated increase in plant cover (or correlated increases in resource quality), whereas in year 5 orthopteran density was not related to cover, diversity or plant %N, but rather to unmeasured effects of P, such as its influence on other aspects of resource quality.

**Conclusions/Significance:**

The marked surprising response to P by orthopterans, combined with a previous observation of P-limitation in lepidopteran herbivores at these sites, suggests that P-mediated effects of food quantity or quality are critical to insect herbivores in this N-P co-limited primary successional system. Our results also support a previous suggestion that the availability of N in these soils is P-limited.

## Introduction

In early successional terrestrial systems, low availability of nitrogen (N) often limits plant productivity [1], [2], [3]. However, in several cases phosphorus (P) or other rock-derived nutrients are known to limit N fixation, especially during inchoate stages of primary succession where P supply depends on the breakdown of calcium apatite minerals [4], [5], [6], [7]. Such systems are considered co-limited by N and P. Insect herbivores may likewise experience nutrient limitation during succession, either through a nutrient limitation to host biomass or through low nutritional value of host plants. This hypothesis has been examined many times in secondary successional systems. For example, Ritchie [8] found that N addition increased grasshopper populations in some abiotic contexts, and Haddad et al [9] found that long-term N addition decreased plant and herbivore diversity but increased plant productivity and herbivore abundance. In one of the few studies of resource limitation to arthropods during primary succession, fertilization of 120-yr-old sites in Hawaii resulted in increased density and biomass of insect herbivores and of spiders [10], [11]. In contrast, Rowe et al. [12] found that although fertilization of mine wastes increased woody plant growth and N concentration, only sap-feeding herbivores were affected. On primary successional sites at Mount St. Helens volcano, lupin growing at high density was lower in leaf %P and %N, and its specialist lepidopteran herbivores responded negatively to low P concentration [13]. We know of no other studies examining resource limitation of arthropods during early primary succession.

Tests of arthropod resource limitation often focus on N because it is commonly limiting to plant productivity and because N limitation appears particularly important for fitness of terrestrial insect herbivores [14], [15], [16]. Thus, tests for bottom-up control of herbivore populations typically manipulate soil N availability, sometimes with dramatic enhancement of herbivore populations [8], [17], [18], [19], [20]. Fewer studies distinguish between N-mediated and P-mediated resource limitation of arthropods, but such investigations are warranted for two reasons. First, recent documentation that primary production in some systems is P-limited [4], [7], [21], [22] raises the possibility that consumers of P-limited plant communities may themselves be P-limited. Second, recent stoichiometric analyses of plant-herbivore interactions suggest the possibility that inadequate P intake in insect herbivores may occur more often than realized. Elser et al. [23] observed that the N:P ratio in plant leaves is, on average, about 21% greater than the N:P of the average terrestrial insect herbivore and concluded that the nutrient demands of these herbivores should be more imbalanced with respect to host P content than to host N. A handful of more direct studies suggest P-limitation of terrestrial insect herbivores [13], [24], [25], [26], [27], [28] or have examined the importance of P in relation to other elements [29], [30], [31], [32], but P-limitation of arthropods is still understudied. Identifying the ecological contexts that may cause P-limitation is an important step toward integrating successional studies with advances in biogeochemistry and trophic ecology.

Here we explore whether major arthropod taxa respond to nutrient addition in a primary successional plant community at Mount St. Helens volcano, and extend our previous study of vegetation response to N and P in order to relate it to arthropod responses. The eruption of 1980 reduced an area of 60 km^2^ to primary successional substrate, where colonization has been dominated by the lupin *Lupinus lepidus* var. *lobbii*, an N-fixing legume that strongly influences the rate and trajectory of succession [33]. Feeding by several guilds of lepidopteran herbivores dramatically affects the population growth and spread of lupins across the landscape [34], [35], [36], [37], [38]. Soil N at these sites has increased substantially but remains in a range inhibitory to primary production [6], [39], [40]. Biomass and cover of non-N fixing plants in this herb-dominated community respond rapidly to N addition, while P addition causes a similar but time-lagged response, probably through its effect on N-fixation by lupin [6]. Leaf-feeding lepidopteran herbivores of lupin are generally absent from high-density lupin patches but abundant in low-density patches, an unusual spatial distribution that appears partially driven by P-limitation to larval growth and survivorship [13].

We hypothesized that N and P addition would have similar, generally positive effects on arthropod abundance because of their positive effects on plant productivity. On the other hand differences in nutritional requirements among arthropod taxa may lead to idiosyncratic responses if fertilization differentially affects the tissue quality or abundance of a preferred host. To test these hypotheses we quantified vegetation and arthropod responses to N and P addition experiments conducted over a five year span. To better understand the mechanisms of N and P effects, we also quantified a correlate of food quality (leaf %N of a representative host), and of N fixation (lupin leaf %N and nodulation).

## Materials and Methods

### Research Area

Our research area was created by the 1980 eruption of Mount St. Helens (46°15′N, 122°10′W), and is located on primary successional pyroclastic deposits and on debris flow deposits on the volcano's north slope (1050–1170 m) (See “Fertilization plots” in Fig. 1 of Gill et al. (2006) for additional site description). The mean annual precipitation (1971–2004) was 224 cm with most precipitation falling in October-May. During the five years of our study (2002–2006), the mean annual temperature was 6.6°C and mean precipitation during the peak growing season (June 15–August 15) was 3.5 cm [6].

**Figure 1:**
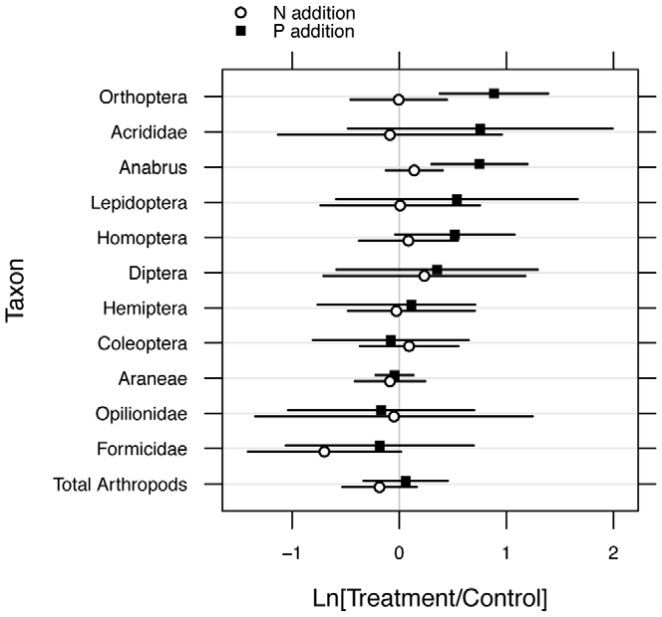
Response of arthropods to N and P addition. Mean log_e_(treatment/control) ±95% CI for taxa sampled in 2004. Squares represent log_e_(P treatment/control), circles represent log_e_(N treatment/control). Lepidoptera are represented by larvae only; Formicidae were primarily *Formica pacifica* and comprised >90% of hymenopterans. Acrididae and *Anabrus* were the most abundant orthopterans.

### Nutrient addition plots

Sets of four 9 m^2^ plots were established at each of eight sites (four on pyroclastic deposits, four on debris flow deposits) in 2002. Plots at each site received a factorial fertilization treatment with N and P in June of each year from 2002 to 2006. To further examine the effect of P, in 2003 eight additional pairs of plots were established on the pyroclastic deposits. One member of each pair received P at the same rates and times as those begun in 2002. We refer to the plots from the two different years as “2002” and “2003” plots or experiments. Both debris flow and pyroclastic soils are rudimentary and susceptible to drought. All sites are in regions of high lupin density referred to in previous papers as “core areas” [36], [37] and had high % cover of low-growing herbaceous plants and moss (primarily Rhacomitrium spp.). Additional description of the plots and the early response of vegetation to fertilization are provided in [6]. The N and P addition rates for the four treatments were: 1) 0 g N and P, 2) N: 7.8 g m^−2^ yr^–1^ as NO_3_NH_4_, 3) P: 10 g m^−2^ yr^−1^ as superphosphate, and 4) both N and P. The amount of N added was ∼10x the annual input of N from atmospheric deposition in the region [6]. The rate of P addition was 5–10% of the recommended rate for legume crops. Observational surveys conducted within these experiments are summarized in Appendix S1.

### Arthropod surveys

In May 2004 one pitfall trap was placed in control, N, and P nutrient addition plots at seven of eight sites of the 2002 experiment, for a total of 21 traps. N+P plots and one site were omitted due to logistical constraints. Traps were dug 10 cm deep, lined with PVC pipe, into which a large plastic cup containing ethylene glycol was inserted. Traps were protected by a piece of plywood supported ∼4 cm above each trap and were closed when traps were inactive. Pitfalls were opened for three ∼one-week periods spanning the most active part of the growing season: June 25^th^ through July 2^nd^, July 13^th^ through July 21^st^ and July 29^th^ through August 7th**.** At the end of each period, contents were transferred to 70% ethanol, sorted to order or family, and counted. Relative abundance of eight arthropod orders were compared among treatments using a linear mixed model regression with *site* treated as a random effect and *treatment* as a fixed effect, with *treatment* nested within *site*. Arthropod count data met regression assumptions after log_e_ transformation. All analyses were conducted using R 2.9.2 [41]. Representative R models and output for most analyses are presented in Appendix S1. Arthropod community analyses were not conducted because pitfall trapping is not an equally effective sampling method for all taxa.

Motivated by strong treatment effects on orthopterans in 2004 (see Results), we further investigated effects of N and P on orthopterans in 2006. Orthopteran density was quantified by counting the number of grasshoppers and Mormon crickets within circular targets of nylon rope (30 cm diameter) [42] in all sites and all treatments of the 2002 experiment and seven sites of the 2003 experiment. Beginning on July 24, 2006, all sites and treatments in the 2002 experiment were surveyed once per day for seven of the next 11 days, Targets were also surveyed in the 2003 plots (P vs. control) at seven sites on four of those days. Surveys were conducted by slowly approaching the plot and counting animals as they left the circular target. Grasshoppers fleeing the circle left the entire plot more than 50% of the time, in effect redistributing them and allowing the opportunity for patch choice prior to the next survey. By design, the observers were unaware of our specific hypotheses regarding nutrient effects on grasshopper abundance. Grasshopper counts were summarized as mean number per day, and analyzed by a linear mixed effects model as described above. 2002 and 2003 experiments were analyzed separately.

### Vegetation

In order to assess effects of fertilization on primary productivity, percent cover was assayed in all plots of both experiments in mid-July from 2004***–***2006. Cover was estimated for each plant species in two 1 m^2^ quadrats within each plot, using the same protocol as in Gill et al. (2006). Plant community response to nutrients was analyzed as effects on % cover of plant functional groups, total % cover of vascular plants, and on the Shannon diversity index. Plants were partitioned into commonly used functional groups [43]: broad-leafed herbaceous species were divided into leguminous and non-leguminous forbs, and grasses and sedges were combined as graminoids. Forbs were dominated by *Hypochaeris radicata* and graminoids were dominated by *Agrostis pallens,* while *L. lepidus* was the only legume. The Shannon diversity index (H) was calculated based on % cover of each species, using the Vegan 1.8–6 package for R [44]. Treatment effects on functional groups were analyzed with a mixed effects model as described for arthropod data, with treatment nested within site to account for non-independence of quadrats, and including an NxP interaction term for the 2002 experiment. Cover was square root-transformed and H was exponentiated (i.e. e^H^, denoted H'), which estimates the effective number of species [45], for all analyses. The 2002 and 2003 experiments were analyzed separately.

### Nodulation and nutrient concentration

To better understand the mechanism by which P affects vegetation, we measured lupin nodulation and leaf %N. Lupin nodule production in the first season of the 2003 experiment was measured in seedlings because earlier work demonstrated much higher rates of N fixation in seedlings at these sites [39], and we expected them to respond more quickly to fertilization. Nodules/cm of root length were counted for 12 seedlings/site (six seedlings/treatment) from the eight 2003 sites, and from several seedlings from each of 10 more recently colonized locations with low lupin density. Log_e_(nodules cm^−1^ +1) was analyzed in a mixed effects model as described above.

Lupin leaf tissue was collected for analysis of nutrient concentration on August 13, 2003, from 6***–***8 plants in each of the 2003 P addition and control plots at 4 sites (N = 54 plants). On August 15, 2005, lupin leaves and culms of *Agrostis pallens* were collected from all plots at all sixteen 2002 and 2003 sites. *A. pallens* was chosen because it was the most common graminoid and occurred in all plots. Plant tissue %N was determined using a Eurovector Elemental Analyzer.

### Arthropod relationship to vegetation

Counts for each arthropod taxon were log_e_-transformed and regressed on H' and % vascular plant cover. Orthopteran counts were also regressed on *Agrostis* %N from 2005 (regressions on *L. lepidus* %N were not conducted because of evidence that these plants were strongly avoided). Although the %N was measured on samples from the end of the 2005 growing season, it should provide a reasonable index of the effects of N and P addition on N concentration [8], [17], [46]. Structural equation modeling (SEM) was conducted in order to partition the effects of N and P addition on orthopteran abundance into direct effects of N and P (which likely act through unmeasured variables such as leaf nutritional value), and indirect effects occurring through the influence of N and P addition on % cover, diversity, and leaf %N. Analyses were conducted using the SEM 0.9***–***19 package for R 2.9.2 [41] and diagrams were drawn using Graphviz (www.graphviz.org). Analyses were based on models specified *a priori* (presented in Results) that included direct effects of site type (pyroclastic flow vs. debris flow) on % cover and diversity. Paths illustrating the effect of site are omitted from the diagrams for clarity of presentation (but illustrated in Appendix S1). %N was dropped from the 2004 model and the NxP interaction term was dropped from the 2006 model because doing so caused large improvements in model fit indices (Appendix S1). Tests of significance and fit are based on log_e_- and square root-transformed variables, but coefficients are reported for back-transformed variables.

## Results

### Arthropod abundance

In 2004, nine orders of arthropods (Coleoptera, Diptera, Hemiptera, Homoptera, Hymenoptera (Formicidae only), Lepidoptera, Orthoptera, Araneae and Opilionidae) were collected in numbers large enough to be analyzed by ANOVA. There was a significant and striking increase in the relative abundance of orthopterans in P addition plots relative to control plots in 2004 (F_1,12_ = 18.8, p = 0.001; Fig. 1). The average increase in orthopteran individuals in P plots relative to controls was 48.7±63.1 animals, a 200% increase relative to controls, while N plots only increased by 1.14±25.7 animals (a 9% increase). Post-hoc tests indicated that P plots attracted significantly more animals than did N (F_1,6_ = 10.2, p = 0.02). Among orthopterans there was a preponderance of Mormon crickets (*Anabrus simplex* (Tettigoniidae), 83%), followed by spur-throated grasshoppers (Acrididae; subfamily Melanoplinae, including *Melanoplus sanguinipes, M. bruneri,* and *M. femurrubrum*) at 12%, band-winged grasshoppers at 2% (subfamily Oedipodinae), with 3% remaining unidentified. When analyzed individually, Acridid grasshoppers and Mormon crickets each showed a significant response to P (Acrididae: F_1,12_ = 10.3, p<0.01, mean increase  = 3.7±3.4 per trap; Mormon crickets: F_1,12_ = 23.1, p<0.001, mean increase  = 38.6 per trap). No significant differences in the abundances of other orders were found among treatments after adjusting for multiple tests, although there was a significant increase in homopterans and lepidopteran larvae in response to P prior to adjustment for multiple tests. Analyses were also conducted at the family level for five taxa (Carabidae, Elateridae, Cicindellidae, Formicidae, and Lycosidae). With the exception of the Elateridae, these are completely or frequently predatory taxa. There was no effect of treatment on these families and no significant correlations between predatory taxa and orthopteran distribution.

**Figure 2:**
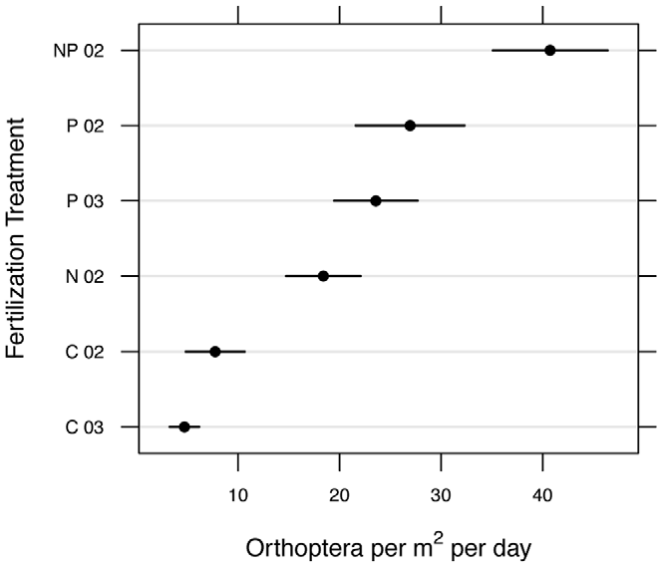
Density of orthopterans (mean ± SE, m^−2^day^−1^) in July 2006, based on visual surveys (see Methods). Treatment labels denote N addition (N), P addition (P), N+P (N & P addition), or control (C), and 02 and 03 denote the experiment start year. Independent variables are on y-axis to facilitate visual comparison of treatments. Corresponding analyses were conducted separately for the two experiments (2002 and 2003).

Our follow-up survey of orthopteran density in 2006, after 5 yrs of fertilization, revealed significant positive effects of both N and P, as determined by counts in circular targets in 2002 plots (N: F_1,21_ = 36.8.1, p<0.0001; P: F_1,21_ = 117.2, p<0.0001; Fig. 2). Post-hoc tests indicated that orthopteran counts were highest in N + P plots, followed by P, then N, then control (all p<0.003). A significant positive effect of P was also observed in the 2003 plots (Fig. 2; F_1,6_ = 30.0, p = 0.002). To summarize, P addition resulted in very highly significant increases in orthopterans in two separate experiments in each of two years despite using two different sampling methods.

### Vegetation

The addition of P and N strongly affected vascular plant cover and species composition in the 2002 experiment in 2004 and 2005, three and four yrs after beginning fertilization. Most notably, both treatments increased forbs (primarily non-native *Hypochaeris radicata*) in both years, while P positively affected lupin in 2004 and N negatively affected lupin (Table 1). A model that included site type (not shown) found no difference in P effects on lupin cover between debris and pyroclastic flow sites. P also positively affected total vascular plant cover in 2005, and both N and P increased diversity in both years. In all cases the effect of N+P combined was additive but not significantly greater than N or P effects (based on post-hoc tests). P also had significant positive effects on lupin in 2004 in the 2003 experiment, followed by increased cover of graminoids and total cover in 2005. These effects of P contrast with a lack of P effects on non-legumes in the first year of fertilization [6]. In 2006, there were few significant effects of treatments on any vegetation categories in either experiment. Our field observations suggest that fertilization attracted gophers (beginning in 2004), whose preferential feeding in the N and P addition plots prevented the N- and P-related increases in cover in 2006 that were observed in most previous years.

**Table 1:** Vegetation response to N and P addition treatments: ANOVA results and means (±SD).

2004	2002 Experiment				2003 Experiment	
Response Variable	Control	N addition	P addition	N+P	Control	P addition
Forbs (except lupin)	13.1 (7.8)	17.8 (8.2) *	21.2 (11.7)***	24.8 (11.7)	8.0 (8.3)	7.1 (8.0)
Graminoids	5.8 (12.2)	7.2 (8.5)	6.2 (7.6)	12.1 (14.4)	5.5 (7.4)	5.7 (6.6)
*Lupinus lepidus*	18.3 (10.7)	8.6 (10.7) ***	25.6 (18.7)*	10.6 (10.6)	30.9 (14.5)	43.3 (16.9)**
Total cover, no moss	41.0 (20.3)	37.2 (18.7)	50.1 (22.2)**	44.1 (19.2)	46.6 (24.9)	56.2 (18.6)
H	1.04 (0.28)	1.28 (0.41)*	1.10 (0.31)*	1.45 (0.34)	1.00 (0.28)	0.97 (0.23)
**2005**						
Forbs (except lupin)	18.6 (15.8)	26.9 (9.9)**	24.9 (11.8)*	36.0 (18.0)	10.4 (7.7)	13.4 (10.0)
Graminoids	11.2 (23.1)	16.3 (19.0)	14.1 (18.0)	19.1 (19.8)	2.6 (3.3)	11.9 (16.1)**
*Lupinus lepidus*	3.4 (3.4)	2.0 (2.9)	1.9 (3.1)	1.3 (2.8)	4.3 (3.5)	2.8 (2.4)
Total cover, no moss	33.1 (21.6)	45.0 (17.4)**	41.5 (20.6)**	55.2 (23.4)	17.2 (7.0)	30.1 (15.0)**
H	0.96 (0.28)	1.22 (0.30)**	1.13 (0.31)*	1.32 (0.30)	1.02 (0.33)	1.20 (0.26)
*Agrostis* %N	0.56 (0.16)	0.91 (0.43)*	0.60 (0.15)	1.0 (0.59)	0.51 (0.15)	0.54 (0.21)
**2006**						
Forbs (except lupin)	21.0 (12.9)	29.5 (19.2)	25.9 (16.1)	31.3 (23.3)	18.5 (7.7)	20.4 (6.2)
Graminoids	13.2 (19.3)	15.3 (16.6)	20.2 (24.6)	26.5 (30.9)	8.2 (4.9)	12.4 (7.5)*
*Lupinus lepidus*	7.4 (7.0)	0.9 (1.5)***	2.6 (6.4)	0.7 (1.6)	12.0 (6.6)	9.1 (7.6)
Total cover, no moss	41.7 (10.6)	45.7 (17.3)	48.7 (19.7)	58.7 (24.0)	38.6 (11.3)	41.8 (14.9)
H	1.15 (0.17)	1.17 (0.24)	1.17 (0.28)	1.23 (0.34)	1.21 (0.16)	1.26 (0.21)

(***: p≤0.001; **: p≤0.01; *: p≤0.05).

P-values for the 2002 (N and P factorial addition) and 2003 (P addition) experiments are from mixed effect ANOVAs (See Appendix S1 for model details). There were no significant N+P interaction terms. Transformations used for analyses: Log_e_ (%N, Forbs, Graminoids), H' ( = e^H^), and (total cover)^1/2^. Bold font highlights significant effects. We report the untransformed values.

### Plant nutrient content

N addition increased tissue %N in the dominant graminoid, *Agrostis pallens*, to 1.69x (average N mass increase  = 0.35%) of control plants collected in August 2005 (Tables 2 & 3; ANOVA for *A. pallens*, all sites: N addition: F_1,19_ = 13.0, p = 0.0007; P addition: F_1,19_ = 1.8, p = 0.14; N x P: F_1,19_ = 0.3, p = 0.47). In contrast, N addition decreased %N in *L. lepidus*, while P addition increased leaf %N on pyroclastic sites by an average of 1.125x in 2005, but not on debris flow sites (Table 2; P x Site type interaction: F_1,16_ = 4.53, p = 0.05; see Appendix S1 for full ANOVA). In 2003 plots, which are also on pyroclastic flows, P addition dramatically increased lupin leaf %N by 0.65%N, 1.31x of the %N in controls (Table 3). P addition also increased the density of nodules on lupin seedlings in 2003 by 53% (Mean ± SE: P addition: 0.55±0.042 cm^−1^; Control: 0.36±0.040 cm^−1^; F_1,87_ = 7.7, p = 0.007). In younger, low density areas, mean nodules cm^−1^  = 0.83, and was unaffected by P addition (analysis not shown).

**Table 2:** Nitrogen concentration in lupin leaves and *Agrostis* stems: 2002 experiment measured in 2005.

	Tissue %N			
	Agpa 2005		Lule 2005	
	Debris	Pyro.	Debris	Pyro.
Control	0.50	0.62	2.48	1.86
P added	0.54	0.65	2.29	2.15^*^
N added	0.67**	1.08*	2.33	1.70**
N+P added	0.78	1.30	2.46	1.68^ **^

(***: p≤0.001; **: p≤0.01; *: p≤0.05; **^x^:** p≤0.08).

“Lule” denotes *Lupinus lepidus* and “Agpa” denotes *Agrostis pallens.*

Data are from four sites each on a debris flow and a pyroclastic flow.

Results based on linear mixed effects models with site as a random effect and df = 1,16. See Appendix S1 for model. Bold font highlights significant results.

**Table 3:** Nitrogen concentration in lupin leaves and *Agrostis* stems: 2003 experiment measured in 2003 and 2005.

	Tissue %N		
	Agpa 2005	Lule 2003	Lule 2005
Control	0.49	2.09	1.86
P added	0.51	2.74***	2.05*

Notation as in Table 2.

Data are from eight sites on a pyroclastic flow.

Results based on mixed effects model (2003 data, df = 1, 49), or on paired t-tests with df = 7 (2005 data). Bold font highlights significant results.

### Arthropod abundance, vegetation, and nitrogen

**Figure 3:**
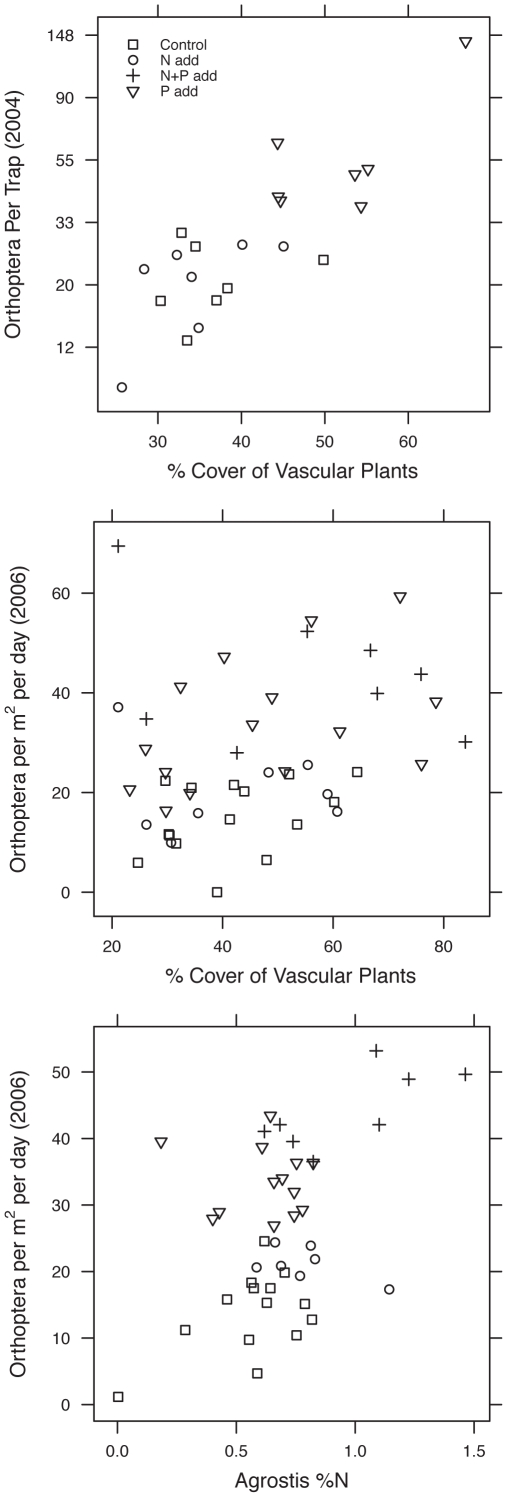
Relationship of orthopteran counts to % cover of vascular plants in 2004 (A) and 2006 (B) and *Agrostis* %N (C) in 2006 after accounting for inter-site variation. **A.** 2002 experiment sampled in 2004; **B &C.** Both experiments, sampled in 2006 (count data); In **Fig. 3C**, note that P addition plots always have more orthopterans than N addition or Control plots with equal or greater leaf N. In all three plots, inter-site variation was removed by regressing each variable on site identity (random effect) and plotting the mean + residuals.

**Figure 4:**
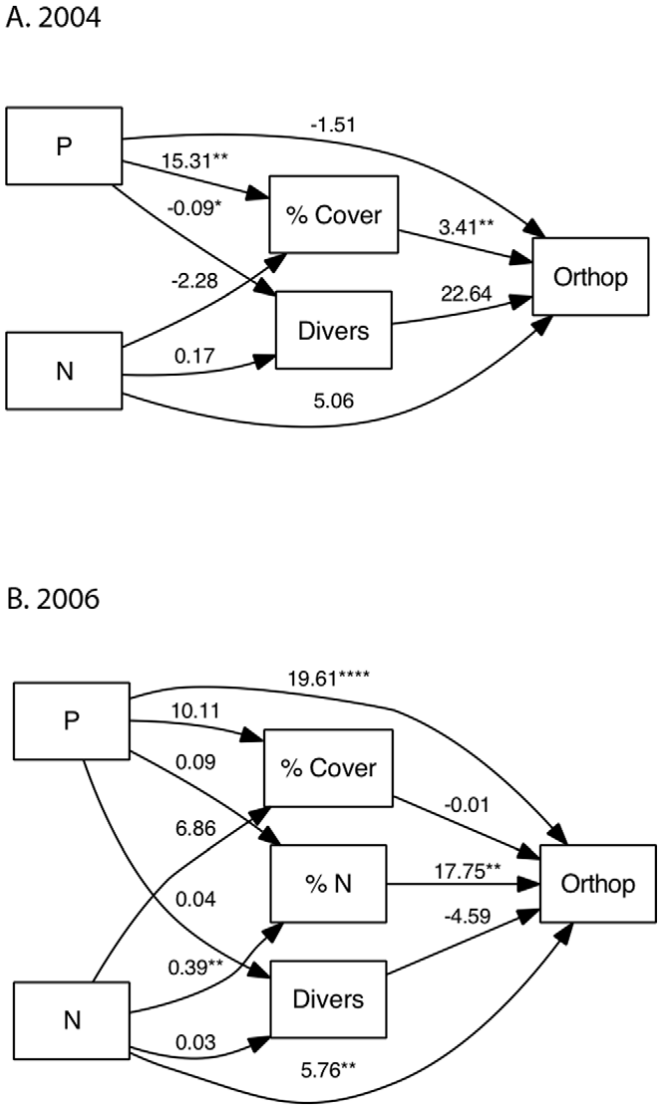
Path diagrams from Structural Equation Models. Results indicate that P affects orthopterans through its effect on cover in 2004, but through an unidentified mechanism such as tissue %P, in 2006. Site variable is omitted from diagrams for clarity (see Appendix S1). Path coefficients in this figure are un-standardized and based on untransformed variables. Significance levels and fit indices (see Results) are based on transformed variables. In 2004, Orthop represents Orthoptera per trap over 27 days of trapping. In 2006, Orthop represents Orthoptera per day per m^2^. *Diversity* is the Shannon Wiener Diversity index (H), % Cover is of vascular plants, and % N is for *Agrostis spp*. Indirect effects are obtained by multiplying paths coefficients, and net effects can be obtained by summing direct and indirect effects. In 2004 for example, the indirect effect of P addition through % cover  = 15.31% *3.41 orthopterans/1% cover change  = 52.2 orthopterans/trap, and the net effect of P through all paths  = 47.2 individuals/trap. (****: p≤0.0001; ***: p≤0.001; *: p≤0.05).

Orthopteran abundance was positively correlated to total % cover of vascular plants in 2004, while arachnids (Araneae) and hemipterans responded positively to vascular plant diversity but not % cover (Table 4; Fig. 3). Based on sequential regression, the positive response of the total number of arthropods to both plant diversity and % cover (Table 4) was driven by the combined influence of at least 5 taxa. In 2006, orthopteran density was more weakly related to cover and diversity (Fig. 3; Table 4). Since these predictors are expected to be influenced by experimental addition of N and P, we employed structural equation models (SEM) to disentangle the effects of N and P addition on orthopterans acting through unmeasured variables from indirect effects acting through these measured predictors. Our SEM was based on an *a priori* causal model (represented in Fig. 4). In addition to the paths shown in Fig. 4, site type (debris flow vs. pyroclastic flow) was allowed to affect all variables except other independent variables (N and P). We omit site from the path diagram for clarity (See Appendix S1 for full diagram). Models for both years fit the data well (2004: Χ^2^_4_ = 7.24, p = 0.13, Bentler-Bonnett NFI  = 0.87; 2006: Χ^2^_6_ = 8.07, p = 0.23, Bentler-Bonnett NFI  = 0.91; See [47] for explanation of SEM fit indices). In 2004, SEM indicates that the strong effect of P on orthopterans was primarily through its effect on percent cover of vascular plants (or unmeasured effects on tissue quality that correlated with % cover). In fact, the addition of P increases orthopteran abundance by 52 animals/trap through its effect on % cover (Fig. 4a). Substituting percent cover of forbs for cover of vascular plants yielded nearly the same model (results not shown). The net effect of P in 2004 was an increase of 47.2 animals/trap, whereas the net effect of N was approximately 1 animal/trap. In 2006, P strongly affected orthopteran density through a direct path, causing an increase of 19.6 animals/day/m^2^ (Fig. 4b). N addition also positively affected orthopterans in 2006, with a net effect of 12.6 animals/day/m^2^, primarily through increasing tissue %N (6.9 animals/day/m^2^).

**Table 4:** Arthropod response to plant community characteristics: Mean abundance (per trap) for different arthropod taxa, and regression coefficients for effects of plant diversity (H') and % Cover^1/2^.

	Mean (SD)^b^	Community Effects	
		H	% Cover
** 2004 Pitfall data**			
** Orthoptera**	68 (87.8)	−8.0***	3.88***
Anabrus	56.1 (92.2)	−9.1	4.00**
Acrididae	7.9 (6.6)	−7.4	0.05
** Diptera** a	24.6 (17.6)	5.42	−014
** Hemiptera**	7.7 (5.3)	14.2**	−0.04
** Homoptera**	4.7 (2.4)	1.9	−0.00
** Opilionidae**	28.1 (17.6)	29.5	−0.72*
** Araneae**	48.1 (16)	39.4	−0.34
** Coleoptera**	22.1 (10.2)	13.9	−0.20
** Formicidae**	78.1	1.3***	0.02
** Total Arthropods** c	219.4 (91.4)	70.2***	3.86**
** 2006 Orthoptera** d,e			
** 2002 plots**	1.65 (1.23)	1.62*	0.023*
** 2003 plots**	0.98 (0.88)	0.58	−0.002

(***: p≤0.001; **: p≤0.01; *: p≤0.05).

Arthropod counts were all log_e_-transformed. Regression coefficients are back-transformed. Bold font highlights significant results.

a None of the Diptera were parasitic tachinids.

b Mean number of individuals/trap.

c Formicidae (ants) omitted.

d Mean number per 0.07 m^2^ target per day; 7 plot pairs for 2003 experiment, 8 plot sets for 2002 experiment.

## Discussion

Our results support Gill et al's (2006) conclusion that primary production on Mount St. Helens' Pumice Plain is limited directly by N availability, but that there is a P-limitation to the supply of N. The effect of P on plant cover lagged behind that of N by 1***–***2 years, but eventually equaled it. With one exception, arthropod taxa showed no significant responses to N or P either individually or in aggregate. However, orthopterans responded dramatically to P addition in both 2004 and 2006, suggesting that P-limitation travels upward to some consumer taxa more strongly than does N.

### Primary production co-limited by N and P

Gill et al's (2006) conclusions of N-limitation and a possible P limitation to N-availability were based on the first three years of data (2002***–***2004) from the same 2002 fertilization experiment analyzed here. To that we have added results from two more years of monitoring (2005***–***2006) and from a second experiment (the 2003 P addition experiment), as well as new analyses of the 2004 data. The hypothesis that P affects the plant community through facilitation of N-fixation is supported by several observations. First is the immediate effect of P on lupin cover in both experiments, followed by an increase in N-limited taxa in the 2002 experiment. P addition increased lupin cover in years 1–3 (Gill et. al. 2006, and Table 1), followed by proportional increases in non-legumes of 25–40% (Table 1) in years 3 and 4 (2004 & 2005). Similarly, in the 2003 experiment there was an initial 50% increase in lupin, followed by a 75% increase in other species. Other findings that support the hypothesis are the P-mediated increases in lupin nodulation, seed production (Gill et. al. 2006), and leaf %N. Interestingly, combined N and P addition exhibited additive effects on non-legume cover while suppressing lupins as much as N addition alone, possibly indicating a direct role for P once N-limitation is removed.

A few other studies have demonstrated P-limitation in early successional systems, although P-limitation is more common on ancient soils [4], [5], [7], [21], [48], [49]. Although primary successional systems initially contain little available P, its availability increases over time through weathering of apatite minerals in the parent material, which at Mount St. Helens contain ∼0.2% P_2_O_5_ [50]. Weathering of P is probably rapid in pumice materials typical of our sites because of its high glass content, surface area, and friability. This release may be accelerated by lupin, which is capable of liberating mineral-bound P through concentrated exudation of organic acids [51].

### Nutrient limitation of consumers

Given the strongly nutrient-limited soils of the Pumice Plain we expected a general arthropod response to both N and P addition. Instead, only a single herbivorous taxon was affected, and it responded primarily to P. P addition dramatically increased the abundance of Orthoptera (primarily Mormon crickets, *Anabrus simplex*, and spur-throated grasshoppers (family Acrididae)) in both 2004 and 2006. While N addition also increased orthopteran density in 2006, its effect was significantly less than that of P. These results contrast with several other studies. For example, Gruner [10], [11] fertilized *Metrosideros*, the dominant tree on 120-yr-old Hawaiian basalt flows that also experience N-P co-limitation and found a more general increase in arthropods at several trophic levels, which was attributable to an increase in host plant quality. Our results also contrast with those of a recent study with a similar design to ours, in which grasshoppers in long term fertilization plots in an N-limited prairie did not increase in abundance with either N or P addition, although fertilization did alter leaf stoichiometry and diet composition [30].

In a separate study at Mount St. Helens we found that the fitness of specialist lepidopteran herbivores feeding on *L. lepidus* was sensitive to natural variation in plant P content and N:P ratio [13]. However, the response of Orthoptera to P is not directly due to an increase in lupin: we found that *Melanoplus spp*. grasshoppers feeding on *L. lepidus* died within one week (unpublished data), and the 2006 response to P occurred despite very low amounts of lupin cover. We suggest that P-limitation to consumers is especially likely on N-fixing plants under P-limited conditions. This hypothesis is congruent with several other studies. For example, P addition in secondary tropical forest significantly increased foliage P and herbivory in leguminous trees growing in young P-limited sites, but not in older, less P-limited sites [31], and the abundance of weevils on *Prosopis*, a leguminous desert shrub, tracked soil P availability [28]. However, our results with Orthoptera also indicate that P-limitation may not be confined to herbivores feeding on N-fixers in these systems.

The lack of response to nutrient addition by most other taxa, including predatory taxa (Table 4) is of interest. A few predators, represented by hemipterans (especially predators in the Nabidae), and spiders (especially in the family Lycosidae) did show a positive response to plant species diversity but not to % cover. This general lack of arthropod response seems surprising since both plant biomass and nutritional quality responded to nutrient addition, but we must add a number of caveats to this conclusion. First, pitfall traps are ineffective at assessing many taxa, especially parasitoid wasps, flies, and lepidopterans. Second, and perhaps most importantly, both the plant response to fertilization and the response by herbivorous arthropods were undoubtedly blunted by the effects of herbivory by vertebrates and orthopterans. Although we were unable to quantify the effect of orthopterans on plants, we estimated a mean orthopteran density of 23 individuals/m^2^ in 2006, (ranging up to 60/m^2^), and these animals visibly affected vegetation at our sites. In addition, plant biomass responses were greatly diminished by dramatic effects of vertebrate herbivores, especially northern pocket gophers (*Thomomys talpoides*). Biomass removal by gophers explains the apparent lack of plant response to N in 2004 and 2006. The invasion of gophers in N and P addition plots, which began in 2004, was striking in its visual impact, with mounded soil dominating the plot and defining the plot boundaries. However, it is important to note that even in 2006, when plant responses to N and P plots were equally blunted by gophers, there was still a highly significant effect of P on orthopteran density.

### Orthopteran response to P

The potential mechanisms through which P addition (and N addition in 2006) affected orthopterans include increased primary production, compositional changes that increase the abundance of preferred hosts, or changes in the nutritional value of host plants. A correspondence analysis of the 2004 data (Appendix S2) indicated that orthopteran numbers increased with increased cover of a large number of herbaceous forbs and grasses, but decreased with the abundance of two other grasses, consistent with a general relationship to primary production. Structural equation modeling (SEM) also indicated that in 2004, the effect of P was primarily through its positive effect on productivity (i.e. percent cover of vascular plants) (Fig. 4). Substituting % cover of forbs for that of vascular plants produced nearly as strong a model, consistent with reports that the three *Melanoplus* species we identified may prefer forbs or legumes over grasses [52], [53]. However, because P addition, % cover, and orthopteran abundance were very highly correlated, effects of P acting through the nutritional quality of hosts could have been masked by the correlation with % cover. Indeed, in 2006 there was no effect of P on percent cover, nor any effects of %cover on Orthoptera, and here the SEM indicated a strong “direct” effect of P on Orthoptera. We surmise that this direct effect most likely occurred through changes in the P-content of host plants. Unfortunately we were unable to measure tissue P content for these samples, but similar studies indicate an increase in tissue %P in grasses and forbs with phosphate addition [30]. We did measure tissue N content and found that the significant effect of N addition in 2006 was entirely through its effect on *Agrostis* %N (which likely is an indicator of %N across grasses and forbs in the plot) (Fig. 4), supporting the hypothesis that Orthoptera are responding to increased nutrient concentration.

The possibility that Orthoptera are responding positively to tissue %P as well as %N is interesting in light of the extensive study of grasshopper and cricket nutritional ecology. Both generalist feeders and relative specialists (for example, graminoid feeders) generally rely on non-optimal food sources, and by actively composing diets from several non-optimal sources they can attain optimal target intake ratios of protein and carbohydrates [30], [54], [55], [56]. Orthoptera also alter diet composition to minimize exposure to defensive compounds or to obtain specific dietary components, such as phenylalanine, which is needed by young nymphs for cuticle formation [57], [58], [59]. Orthoptera are highly mobile and clearly capable of discriminating and choosing among food sources of different quality. However, nearly all of these nutritional studies have focused on protein and carbohydrates, and none have included explicit study of nucleic acids, the principal biological source of P (but see [60], who found protein, but not P affected grasshopper performance). Interestingly, a recent examination of field crickets (*Gryllus texensis*) found surprising levels of variation in carcass nutrient composition: 400% variation in %P but only 50% variation in %N [61]. Because ribosomal RNA represents the principal pool of tissue P, this suggests that variation in %P could be linked to growth rate [23]. In a companion study, *G. texensis* males exhibited a correlation between carcass %P and long distance mate signaling, a sexually selected trait [62]. These results support the possibility that dietary P is at times limiting to orthopteran fitness. Several other studies on specialist terrestrial insect herbivores also suggest that P may be more limiting than commonly thought [13], [24], [28]. Our results clearly warrant further investigation into P limitation of Orthoptera at Mount St. Helens. In addition, our results suggest that investigating spatiotemporal variation in N and P availability will lead to a better understanding of consumer dynamics in primary succession.

## Supporting Information

Appendix S1 Supplementary information regarding sampling and statistical analyses. This file contains supplementary information regarding sampling and analyses. Section 1 describes the sampling patterns for vegetation, arthropods, and other variables within the fertilization plots. Section 2 provides representative R models and results for the analyses described in the main text. Section 3 contains full SEM models and additional information regarding model choice.
(0.11 MB DOC)

Appendix S2 Detrended correspondence analysis of vegetation data. Detrended correspondence analysis of Mount St. Helens vegetation data from control, N addition, and P addition plots. Plots received N and P additions in 2002, 2003, and 2004; quadrats were assessed for % cover in 2004. Species abbreviations are in Appendix S1 Table 1. The best species spread in ordination space was achieved without data transformations and without down-weighting the influence of rare species. A second matrix of grasshopper abundance was mapped onto the vegetation data matrix. Thus, a “gh” vector shows the direction of increased grasshopper abundance (to the right) in the plots in 2004. PC-ORD was used for analysis and graphics (MJM Software 2002).
(0.05 MB DOC)
