# Supplementary material for: N-P Co-Limitation of Primary Production and Response of Arthropods to N and P in Early Primary Succession on Mount St. Helens Volcano
Source: PLoS One. 2010 Oct 26;5(10):e13598. doi: 10.1371/journal.pone.0013598 (PMC2964294; doi:10.1371/journal.pone.0013598)
Supplement: Appendix S1 — Supplementary information regarding sampling and statistical analyses. This file contains supplementary information regarding sampling and analyses. Section 1 describes the sampling patterns for vegetation, arthropods, and other variables within the fertilization plots. Section 2 provides representative R models and results for the analyses described in the main text. Section 3 contains full SEM models and additional information regarding model choice. (0.11 MB DOC) [file pone.0013598.s001.doc]

**Appendix S1.**

**Section 1. Summary of experimental design and sampling.** First row shows the design for the two nutrient addition experiments. The remaining rows provide information about what was sampled in those plots. Treatments were repeated at each site. C= control, N = N addition, P = P addition

|  | **2002 Experiment** | | **2003 Experiment** | |
| --- | --- | --- | --- | --- |
|  | **Treatments** | **Sites** | **Treatments** | **Sites** |
| Fertilization treatments, 2002-2006 | C, N, P, N+P | 8 | C, P | 8 |
| Arthropod survey, 2004 | C, N, P | 7 | -- | -- |
| Orthopteran survey, 2006 | C, N, P, N+P | 8 (7 days) | C, P | 7 (4 days) |
| Vegetation surveys, 2004-2006 | C, N, P, N+P | 8 | C, P | 8 (7 in 2006) |
| Nodulation, 2003 | -- | -- | C, P | 8 |
| Lupin %N, 2003 | -- | -- | C, P | 4 |
| Lupin and Agrostis %N, 2005 | C, N, P, N+P | 8 | C, P | 8 |

**Section 2. Summary of statistical models.** The information below contains the R commands and models used for the analyses in tables 1-3. Also included is representative portions of summary output that show the sample size and degrees of freedom.

1. **Analysis of treatment effects on vegetation characteristics, 2004-2006.** For 2002 experiment, vegetation was quantified in two 1m^2^ quadrats per plot in all four treatments at all 8 sites from 2004-2006. For 2003 experiment, it was quantified in both treatments at all 8 sites (except in 2006, when one site was accidentally omitted). Unit of analysis was the quadrat, with quadrat nested within site to account for the non-independence of quadrats within plots).

   A model of the following form was run in R for each of the dependent variables:

lme( fixed = sqrt(cover) ~ P + N + N:P, random = ~ 1 | quadrat %in% site.no, data= veg.2005, subset=year==”2002”)

Yielding the following ANOVA table:

numDF denDF F-value p-value

(Intercept) 1 32 56.35516 <.0001

N 1 21 9.01790 0.0068

P 1 21 4.79601 0.0400

N:P 1 21 0.04206 0.8395

Number of Observations: 64

Number of Groups: site: 8, treat %in% site: 32

For the 2003 experiment, a representative model and results were as follows:

lme( fixed = sqrt(cover) ~ P, random = ~ 1 | treat %in% site.no, data= veg.2005, subset=year==”2003”)

numDF denDF F-value p-value

(Intercept) 1 16 428.8981 <.0001

P 1 7 12.3150 0.0099

Number of Observations: 32

Number of Groups: site: 8, treat %in% site: 16

1. **Treatment and site type effects on *L. lepidus* leaf %N**. The positive effect of P addition on lupin %N occurred only on pyroclastic flow sites. This is reflected in a significant P x site type interaction in the following ANOVA from a linear mixed effects model with site as a random effect. The significant three way interaction reflects a more negative effect of N addition in the presence of P on pyroclastic flows. Other terms were dropped without decreasing model fit.

tmp <- lme(pctN ~ P + site.type + site.type :P + site.type:P:N, random= ~1|site.no, data=fert.lule)

numDF denDF F-value p-value

(Intercept) 1 16 622.2077 <.0001

Padd 1 16 0.6835 0.4205

substr 1 6 10.5853 0.0174

Padd:substr 1 16 4.5309 0.0492

substr:Nadd 2 16 8.5614 0.0030

Padd:substr:Nadd 2 16 3.4220 0.0579

1. **Analysis of treatment effects on 2004 arthropod abundance data.** We used pitfall traps to sample arthropods in P addition, N addition, and control plots at each of 7 sites. For each taxon a linear mixed effects model of the following form was run in R:

lme( fixed = log(Orthoptera) ~ N+P, random = ~ 1 | site.no, data=bugs04.dat)

Yielding the following ANOVA table:

Analysis of Variance Table

numDF denDF F-value p-value

(Intercept) 1 12 36.88828 0.0001

P 1 12 18.76011 0.0010

N 1 12 0.02814 0.8696

Number of Observations: 21

Number of Groups: 7

1. **Analysis of treatment effects on 2006 orthopteran density.** In 2006 the dependent variable is orthopera/m^2^/day. In 2006 orthopteran density was sample in all four treatments of the **2002 experiment**, and the following mixed effect model was run in R

lme( fixed = log(Orthoptera) ~ N*P, random = ~ 1 | site.no, data= orth06.dat, subset=year=="2002")

Yielding the following ANOVA table:

numDF denDF F-value p-value

(Intercept) 1 21 62.94198 <.0001

N 1 21 56.49445 <.0001

P 1 21 172.95461 <.0001

NP 1 21 0.75396 0.395

Number of Observations: 32

Number of Groups: 8

In 2006 orthopteran density was also quantified in both treatments of the 2003 experiment (one site was omitted due to erosion), and analyzed using the following model:

lme( fixed = log(Orthoptera) ~ P, random = ~ 1 | site.no, data=orth06.dat, subset=year=="2003")

Yielding the following ANOVA table:

numDF denDF F-value p-value

(Intercept) 1 6 35.13128 1e-03

P 1 6 62.80751 2e-04

Number of Observations: 14

Number of Groups: 7

1. **Analysis of H’ and % cover on arthropod taxa in 2004.** For each taxon, the following command produced a linear mixed effects model. A representative ANOVA table is also shown. Exp(H) = e^H^.

**lme(log(Orthoptera) ~ exp(H) + pct.cover, random=~ 1 | site.no, data=jnk2)**

numDF denDF F-value p-value

(Intercept) 1 12 113.57087 <.0001

exp(H) 1 12 21.48687 6e-04

pct.cover 1 12 32.78533 1e-04

Number of Observations: 21

Number of Groups: 7

**Section 3. Complete representation of path model illustrated in Fig. 4 and notes on model selection.** Path coefficients in this figure are un-standardized and based on untransformed variables. Significance levels and fit indices (see Results) are based on transformed variables. In 2004, Orthop represents Orthoptera per trap over 27 days of trapping. In 2006, Orthop represents orthoptera per day per m^2^. Divers is the Shannon Diversity index (H). Rather than include a variable for each site, Site represents debris flow (0) or pyroclastic flow (1). (****: p < 0.0001; ***: p < 0.001; *: p< 0.05).

**2004.**


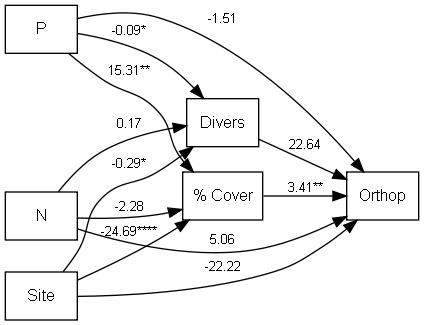


**B. 2006.**

**
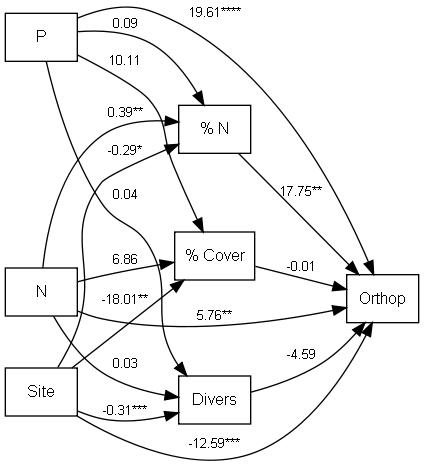
**

**Additional notes on SEM model selection.** Although we used a model specified a priori, we found it necessary to remove Agrostis %N from the 2004 model and the NxP interaction term from the 2006 model in order to obtain an acceptable model fit.

1. *Percent N.* Agrostis %N (shown in the 2006 model) was measured in 2005, but based on other studies of N addition on tissue N for grasses and non-legume forbs, it should provide a reasonable indication of effects of N addition on tissue N in 2004 and 2006. However, for the 2004 data set, there were no indications of a significant effect of %N, and in fact we were unable to achieve an acceptable model fit with %N in the model.
2. *Interaction terms.* The 2006 model included data from all four treatments of the factorial N and P addition, so it is possible to fit an interaction term in the model. We deleted paths involving NxP in a stepwise fashion, and compared the resulting models at each step using a likelihood ratio test. Deleting those paths never worsened the fit of the model, and in fact we were unable to obtain a model with acceptable fit until NxP was completely deleted from it.
