# Supplementary material for: N-P Co-Limitation of Primary Production and Response of Arthropods to N and P in Early Primary Succession on Mount St. Helens Volcano
Source: PLoS One. 2010 Oct 26;5(10):e13598. doi: 10.1371/journal.pone.0013598 (PMC2964294; doi:10.1371/journal.pone.0013598)
Supplement: Appendix S2 — Detrended correspondence analysis of vegetation data. Detrended correspondence analysis of Mount St. Helens vegetation data from control, N addition, and P addition plots. Plots received N and P additions in 2002, 2003, and 2004; quadrats were assessed for % cover in 2004. Species abbreviations are in Appendix S1 Table 1. The best species spread in ordination space was achieved without data transformations and without down-weighting the influence of rare species. A second matrix of grasshopper abundance was mapped onto the vegetation data matrix. Thus, a “gh” vector shows the direction of increased grasshopper abundance (to the right) in the plots in 2004. PC-ORD was used for analysis and graphics (MJM Software 2002). (0.05 MB DOC) [file pone.0013598.s002.doc]

**Appendix S2.** Detrended correspondence analysis (Hill and Gauch 1980) of Mount St. Helens vegetation data from control, N addition, and P addition plots. Plots received N and P additions in 2002, 2003, and 2004; quadrats were assessed for % cover in 2004. Species abbreviations are in Appendix S1 Table 1 The best species spread in ordination space was achieved without data transformations and without down-weighting the influence of rare species. A second matrix of grasshopper abundance was mapped onto the vegetation data matrix. Thus, a "gh" vector shows the direction of increased grasshopper abundance (to the right) in the plots in 2004. PC-ORD was used for analysis and graphics (MJM Software 2002).


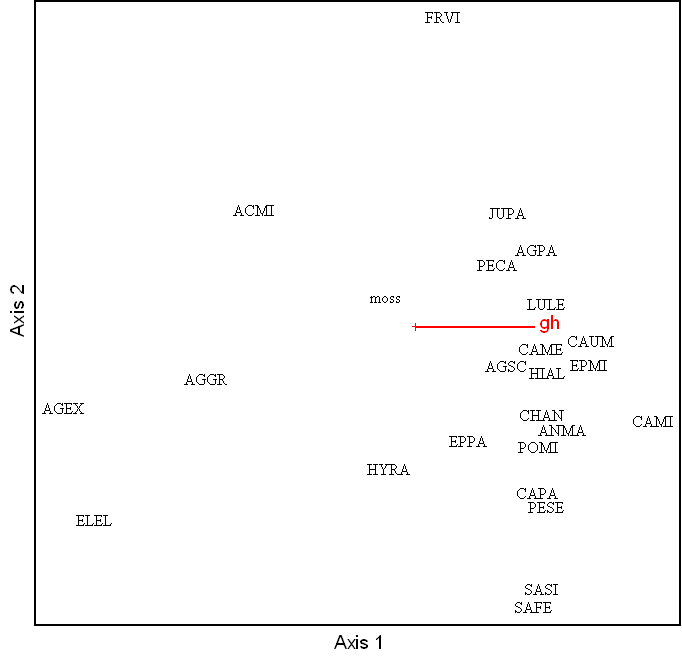


**Appendix S1 Table 1.** Species present in the control, nitrogen and phosphorus plots on the Pumice Plain of Mount St. Helens. Taxonomy follows USDA Plants Database (<http://plants.usda.gov/index.html>). E = non-native species.

| ACMI | *Achillea millefolium* |
| --- | --- |
| AGEX | *Agrostis exerata* |
| AGGR | *Agoseris grandiflora* |
| AGPA | *Agrostis pallens* |
| AGSC | *Agrostis scabra* |
| ANMA | *Anaphalis margaritacea* |
| CAME | *Carex mertensii* |
| CAMI | *Castilleja miniata* |
| CAPA | *Carex pachystachya* |
| CAUM | *Calyptridium umbellatum (Spragea umbellatum)* |
| CHAN | *Chamerion angustifolium (Epilobium angustifolium)* |
| ELEL | *Elymus elymoides (Sitanion hystrix)* |
| EPMI | *Epilobium minutum* |
| EPPA | *Epilobium paniculatum* |
| FRVI | *Fragaria virginiana* |
| HIAL | *Hieracium albiflorum* |
| HYRA | *Hypochaeris radicata* (E) |
| JUPA | *Juncus parryi* |
| LULE | *Lupinus lepidus* |
| moss | moss |
| PECA | *Penstemon cardwellii* |
| PESE | *Penstemon serrulatus* |
| POMI | *Polygonum minimum* |
| SAFE | *Saxifraga ferruginea* |
| SASI | *Salix sitchensis* |
